# Metabolic connectivity as a predictor of surgical outcome in mesial temporal lobe epilepsy

**DOI:** 10.1002/epi4.12853

**Published:** 2024-01-03

**Authors:** Ondřej Strýček, Pavel Říha, Martin Kojan, Zdeněk Řehák, Milan Brázdil

**Affiliations:** ^1^ Brno Epilepsy Center, First Department of Neurology, St. Anne's University Hospital and Faculty of Medicine Masaryk University, Member of ERN‐EpiCARE Brno Czech Republic; ^2^ Central European Institute of Technology (CEITEC) Masaryk University Brno Czech Republic; ^3^ Department of Nuclear Medicine Masaryk Memorial Cancer Institute Brno Czech Republic

**Keywords:** mesial temporal lobe epilepsy, metabolic connectivity, positron emission tomography

## Abstract

**Objective:**

The study investigated metabolic connectivity (MC) differences between patients with unilateral drug‐resistant mesial temporal lobe epilepsy (MTLE) with hippocampal sclerosis (HS) and healthy controls (HCs), based on [18 F]‐fluorodeoxyglucose (FDG)–PET data. We focused on the MC changes dependent on the lateralization of the epileptogenic lobe and on correlations with postoperative outcomes.

**Methods:**

FDG–PET scans of 47 patients with unilateral MTLE with histopathologically proven HS and 25 HC were included in the study. All the patients underwent a standard anterior temporal lobectomy and were more than 2 years after the surgery. MC changes were compared between the two HS groups (left HS, right HS) and HC. Differences between the metabolic network of seizure‐free and non‐seizure‐free patients after surgery were depicted afterward. Network changes were correlated with clinical characteristics.

**Results:**

The study showed widespread metabolic network changes in the HS patients as compared to HC. The changes were more extensive in the right HS than in the left HS. Unfavorable surgical outcomes were found in patients with decreased MC within the network including both the lesional and contralesional hippocampus, ipsilesional frontal operculum, and contralesional insula. Favorable outcomes correlated with decreased MC within the network involving both orbitofrontal cortices and the ipsilesional temporal lobe.

**Significance:**

There are major differences in the metabolic networks of left and right HS, with more extensive changes in right HS. The changes within the metabolic network could help predict surgical outcomes in patients with HS. MC may identify patients with potentially unfavorable outcomes and direct them to a more detailed presurgical evaluation.

**Plain Language Summary:**

Metabolic connectivity is a promising method for metabolic network mapping. Metabolic networks in mesial temporal lobe epilepsy are dependent on lateralization of the epileptogenic lobe and could predict surgical outcomes.

**Key points**

- There are more changes in the metabolic network in right hippocampal sclerosis than in left hippocampal sclerosis.
- Metabolic connectivity (MC) may be a predictive factor in surgical outcomes.
- Decreased MC of operculo‐insular regions with the hippocampi positively correlates with surgery failure.

## 1 INTRODUCTION

Epilepsy is considered to be a network disease, in which networks are significantly involved in the propagation and possible generation of abnormal epileptic activity. Approximately 30% of patients are resistant to anti‐seizure medications (ASMs).^1^ Patients with drug‐resistant epilepsy (DRE) may benefit from epilepsy surgery as an important treatment option. In such cases, it is essential to precisely define an epileptogenic network (EN).

Mesial temporal lobe epilepsy (MTLE) with hippocampal sclerosis (HS) is a common DRE in adults.^2^ However, even after the removal of a significant portion of the epileptogenic hippocampus, only 57% of patients achieve complete seizure freedom (outcome ILAE 1).^3^ One possible reason for the failure of epilepsy surgery is the existence of an EN extending beyond the pathological hippocampus, which may continue to generate seizures after surgery.^4^ These networks can be studied from different perspectives, one of which is metabolic connectivity (MC). The analysis of MC is based on [18 F]‐fluorodeoxyglucose (FDG) positron emission tomography (PET) data. PET is used as a standard in the preoperative evaluation of patients with DRE and is therefore performed in the majority of patients with HS. In HS, typical hypometabolism is seen in the epileptogenic hippocampus, but metabolic changes are also present in other, more remote (extratemporal) areas. These temporal and extratemporal metabolic changes may correlate with the surgical outcome.^5^, ^6^, ^7^ Ipsilateral mesial frontal and perisylvian hypometabolism in the right HS and contralateral fronto‐insular hypometabolism with posterior white matter hypermetabolism in the left HS correlate with poorer surgical outcomes (not seizure‐free).^7^ There is also evidence of changes in MC in patients with HS.^8^, ^9^ However, the effect on the epileptogenic side in MC has not been investigated in any of the previous studies. It is known from studies on functional connectivity (FC) with EEG and functional magnetic resonance imaging (fMRI), that there are major differences between left and right HS and that it is beneficial to study them separately. In general, the FC changes are more extensive in the left HS.^10^, ^11^ Unlike, structural network abnormalities within the temporolimbic network could be more pronounced in the right HS.^12^ The reason for such contradictory findings and the clinical significance of lateralization of structural and functional network changes are still unclear. According to some FC studies, network changes may predict post‐surgical outcomes.^13^, ^14^ So far, only one study focused on the correlation between metabolic network changes and surgical outcomes. The study reported that non‐seizure‐free patients had significant differences in global metabolic networks compared to seizure‐free patients (assessed 1 year after surgery).^15^ As this study demonstrated that MC is a global and non‐specific predictor of surgical outcome, we focused our analysis on regions of interest (ROIs). ROIs included the adjacent cortex where HS seizures typically propagate, most commonly described as hypometabolic in patients with HS, and parts of the traditionally assumed epileptogenic network in HS.^5^, ^7^, ^16^, ^17^, ^18^ The study aimed to explore MC changes in patients with HS and identify changes that could serve as potential surgical outcome predictors. These ROIs could potentially serve as targets for a more precise preoperative evaluation in selected patients, such as those receiving intracerebral EEG.

## 2 METHODS

### 2.1 Participants

In the patient group, we included data from the database of patients with drug‐resistant HS who underwent antero‐medial temporal lobe resection (AMTR) at the Brno Epilepsy Center between 2016 and 2020. The diagnosis was based on presurgical evaluation including long‐term video‐EEG monitoring, neuropsychological assessment, MRI, and FDG–PET. MRI scans were performed with a Siemens 3‐T MRI scanner. There were 47 patients (31 female, 16 male). The mean age of the patients was 40.5 (±11.3) years. None of the subjects had major visual or cognitive impairment, except for a memory deficit reflecting mesiotemporal impairment. All patients had histopathological evidence of HS; three of them had associated focal cortical dysplasia in the temporal pole. Only patients with a follow‐up of more than 2 years were considered for analysis. Patients were categorized as seizure‐free (ILAE 1) or non‐seizure‐free (ILAE 2–5) based on the outcome. Patient characteristics are listed in Table 1: Clinical data collected included age at epilepsy onset, epilepsy duration, and age at the time of examination. The control group (HC) included data from the database of patients with oncology. This group comprised 25 individuals (14 female, 11 male) who underwent a standard FDG–PET examination as part of the oncology screening protocol; all of them had normal PET findings. The mean age of the HC at the time of imaging was 53 (±18) years. The HC had no neurological or psychiatric disorders and had no pathological findings on their MRI scans. They were not taking any medications that affect brain metabolism.

**TABLE 1:** Clinical characteristics of patients according to lesional side (left vs. right HS).

Clinical characteristics	HS	Left HS	Right HS	Left versus right (*p* value)
Number	47	26	21	
Sex, n males/females	16/31	13/13	3/18	**0.0140**
Age at epilepsy onset (mean ± SD)	13.1 ± 10.4	13.2 ± 10.9	12.8 ± 9.9	0.9487
Age at operation (mean ± SD)	40.5 ± 11.3	38.4 ± 10.9	43.2 ± 11.45	0.1541
Epilepsy duration (mean ± SD)	27.5 ± 15.3	25.1 ± 15.2	30.4 ± 15.3	0.1774
Seizures frequency, n/month	8.0 ± 8.9	9.0 ± 9.3	6.9 ± 8.4	0.0827
FBTCS, n of patients (%)	25 (53%)	14 (54%)	11 (52%)	1.0000
Epilepsy history, n of patients (%)				
Familial epilepsy	3 (6%)	2 (8%)	1 (5%)	1.0000
Early brain injury	15 (32%)	8 (31%)	7 (33%)	1.0000
Febrile seizures	11 (23%)	6 (23%)	5 (24%)	1.0000
ASM (mean ± SD)	2.3 ± 0.7	2.4 ± 0.6	2.3 ± 0.7	0.9529
Outcome, n of patients (%)				
ILAE 1	26 (55%)	14 (54%)	12 (57%)	1.0000
ILAE non‐1	21 (45%)	12 (46%)	9 (43%)	1.0000

*Note*: Significant differences are depicted in bold.

Abbreviations: ASM, anti‐seizure medication; FBTCS, focal to bilateral tonic–clonic seizure; HS, hippocampal sclerosis; n, number; SD, standard deviation; y, years.

### 2.2 Clinical and MR data characteristics

For the assessment of structural MRI data MRI, the approach outlined by Chassoix et al.^24^ was employed. Two trained neurologists, who were blinded to clinical data, visually analyzed the patients' MRI scans to determine the extent of hippocampal atrophy (mild, moderate, or severe), temporal lobe atrophy, and signal changes (or blurring between gray and white matter) in the temporal pole. The same methodology was used for defining of electroclinical patterns of patient's seizures: pure mesial group (M) without evidence of early spread beyond the temporal lobe; widespread mesio‐lateral group (WML) with wide spread of seizures to both anterior and posterior lateral temporal and perisylvian areas; antero‐mesial group (AML) with anterior spread (involving the anterior lateral temporal cortex and insulo‐fronto‐opercular areas and bitemporal group (BT) with early contralateral temporal spread. For more details on semiological classification see Chassoix et al. study.^24^ The classification of electroclinical patterns was based on video‐EEG findings (both semiological and electrophysiological) and stereo‐EEG data when available. For practical reasons, patients from the M group were compared with patients from other groups (WML, AML, BT).

Differences in clinical data between study groups (left HS vs. right HS, and ILAE1 vs. ILAE non‐I) were analyzed using a Mann–Whitney test for numerical variables (age at onset, age at surgery, disease duration, seizure frequency, and number of ASMs). The Fisher exact test was used for binary variables such as sex, history of focal to the bilateral tonic–clonic seizures (FBTCSs), history of early brain trauma, febrile seizures, familial epilepsy, and outcome. The Chi‐Square test was used for nominal variables such as electroclinical pattern, HS degree, temporal atrophy, and polar signal abnormality with a significance level of *P* < 0.05.

### 2.3 PET acquisition and processing

Patients were imaged as outpatients in the interictal state. Subjects were prepared by fasting for 6 hours before the scan and resting in a quiet, darkened room for 50–60 minutes after FDG administration. EEG monitoring was not performed during the scan and patients were asked to report any seizures experienced on the day of the scan. The dose of FDG administered was 170–200 MBq per subject with no weight differentiation. The PET images were acquired using a Siemens mCT Flow PET/CT scanner in 3‐D mode using the “Brain” protocol. The intrinsic spatial resolution of the scanner was 6.3 mm at full width at half maximum (FWHM) 1 cm from the center of the FOV, and 6.7 mm at FWHM 10 cm from the center of the FOV. The emission acquisition time in 3‐D mode was 10 minutes. Forty‐seven tomographic slices with a 3 mm slice thickness were reconstructed with a 128 × 128 iteration matrix with 6 iterations and 16 subsets, and a 6 mm FWHM Gaussian filter was applied. Spatial pre‐processing and statistical analysis were performed using SPM12 (Wellcome Department of Cognitive Neurology, Institute of Neurology, University College London, UK). All the FDG–PET images were spatially normalized into the standardized stereotactic Montreal Neurological Institute space using our in‐house FDG–PET template. This template was created following instructions introduced by Soma et al. and described in a study by Kojan et al.^19^, ^20^ A three‐dimensional isotropic Gaussian kernel with 8 mm FWHM was used for smoothing all spatially normalized images. The regional standard uptake value (SUV) ratio was then calculated using the cerebellum as the reference area according to the following equation: SUVR = (SUV ROI)/(SUV total cerebellum).^21^ The subject's regional SUV ratio was extracted for 20 ROIs based on an automated anatomical labeling atlas 1 (AAL1).^22^

The procedure described by Huang et al. was used to calculate the individual MC matrix.^23^ Briefly, individual MC is calculated by combining group connectivity and an individual weighting matrix. The PET image is used to extract signals from selected areas based on the atlas, from which the mean metabolic activity and group correlation in the healthy control group are derived. The individual MC is obtained by combining the group connectivity with the individual weighting matrix, which is determined based on the difference between the individual metabolic activity and the mean group activity.

Individual connectivity matrices were compared between HC, left HS, right HS, seizure‐free HS, and non‐seizure‐free HS groups using a two‐sample *t*‐test. Sex and age were included as nuisance variables. To facilitate comparison and interpretation of results, patient data were unilateralized, i.e., images of patients with right HS were flipped on the right–left axis, while the images of patients with left HS remained unchanged. In the unilateralized data, the hippocampus with the lesion was consistently on the left side. Multiple testing correction via false discovery rate (FDR) was performed in all the results.

To examine the correlation between clinical factors, including the epilepsy onset, duration, frequency of seizures, electroclinical patterns, the degree of hippocampal sclerosis (HS), temporal atrophy, and polar signal abnormalities, with MC values that were significantly different in the ILAE‐1 and ILAE‐non1 groups identified in the previous analysis, the Spearman correlation coefficient was employed.

## 3 RESULTS

### 3.1 Clinical, electroclinical, and neuroimaging data characteristics

#### 3.1.1 HS versus HC

There was a significant difference in age between the HC and HS groups (*P* = 0.0024).

#### 3.1.2 Left HS versus right HS

There were significantly more males in the left HS group and significantly more females in the right HS group. There were no other differences between the left HS and right HS groups in clinical characteristics (mean age at epilepsy onset and at surgery, age at onset, seizure duration, seizure frequency, history of FBTCS, history of early traumatic brain injury, febrile seizures, family history of epilepsy, and the number of ASMs). Details are shown in Table 1.

#### 3.1.3 Seizure‐free versus non‐seizure‐free patients

Fifty‐five percent of the patients were seizure‐free (ILAE 1). In terms of structural data there was a correlation between the degree of HS and worse outcome, but with a low level of statistical significance (*P* = 0.0438). There were no other differences between the seizure‐free (ILAE 1) and not‐seizure‐free (ILAE 2–5) groups regarding the clinical data, electroclinical patterns, presence of temporal atrophy, or temporal polar abnormalities. Details are shown in Table 2.

**TABLE 2:** Clinical characteristics of patients according to the surgical outcome.

Variable	ILAE 1	ILAE non‐1	ILAE 1 versus non‐1 (*p* value)
Males, n males/females	9/17	7/14	1.0000
Age at epilepsy onset (mean ± SD)	12.2 ± 10.1	14.1 ± 10.9	0.6144
Age at operation (mean ± SD)	40.2 ± 10.5	41.0 ± 12.45	0.8053
Epilepsy duration (mean ± SD)	28.0 ± 14.0	26.9 ± 17.1	0.7400
Seizures frequency, n/month	8.7 ± 7.8	7.3 ± 10.2	0.1427
FBTCS, n of patients (%)	15 (58%)	10 (48%)	0.5643
Epilepsy history, n of patients (%)			
Familial epilepsy	3 (12%)	0 (0%)	0.2424
Early brain injury	9 (35%)	6 (29%)	0.7583
Febrile seizures	6 (23%)	5 (24%)	1.0000
ASM (mean ± SD)	2.4 ± 0.6	2.3 ± 0.7	0.6794
Electroclinical pattern			
M	5	2	0.7367
WML	4	4	
BT	5	3	
AML	12	12	
HS degree			
0	4	1	
1	8	2	
2	7	14	
3	7	4	**0.0438**
Temporal atrophy			
−	13	11	0.8710
+	13	10	
Polar signal abnormality			
−	15	14	0.5292
+	11	7	

*Note*: Electroclinical charateristic of the patients are based on see Chassoix et al. study.^24^ Structural data characteristics of the patients based on MRI scans using procedure of the same study. Significant differences are depicted in bold.

Abbreviations: AML, antero‐mesial group; ASM, anti‐seizure medication; BT, bitemporal group; FBTCS, focal‐to‐bilateral tonic–clonic seizure; HS, hippocampal sclerosis; M, mesial group; n, number; SD, standard deviation; WML, widespread mesio‐lateral group; y, years.

### 3.2 Changes in MC in HS compared with HC

Compared to HC, patients with HS had significantly decreased MC in both temporal and extratemporal areas. No increase in MC was observed. In the temporal areas, decreased MC was found in the mesiotemporal structures, temporal pole, and temporal lateral and inferolateral cortex (inferior, middle, and superior temporal gyrus). In the mesiotemporal structures, the MC was decreased between both hippocampi and parahippocampal gyri. The most significant changes in MC were found in the ipsilesional (on the same side as the lesional hippocampus) inferolateral temporal cortex (inferior temporal gyrus), which showed decreased MC in both insulas, orbitofrontal cortices, and ipsilesional mesiotemporal structures. The MC of this area showed even more changes than the lesional hippocampus. Decreased MC was also found in the ipsilesional temporal pole, which decreased particularly in contralesional areas (mesiotemporal, temporal pole, and insula).

From the extratemporal areas, the majority of changes were seen in the orbitofrontal cortex, particularly ipsilesional. The ipsilesional orbitofrontal cortex showed decreased MC, particularly in the contralesional (contralateral to the lesional hippocampus) insula and the temporal inferolateral and lateral cortex (inferior, middle, and superior temporal gyrus). The contralesional orbitofrontal cortex showed decreased MC prevalently with the ipsilesional temporal lateral cortex. Finally, the operculo‐insular regions showed most MC changes in both insulas.

**FIGURE 1:**
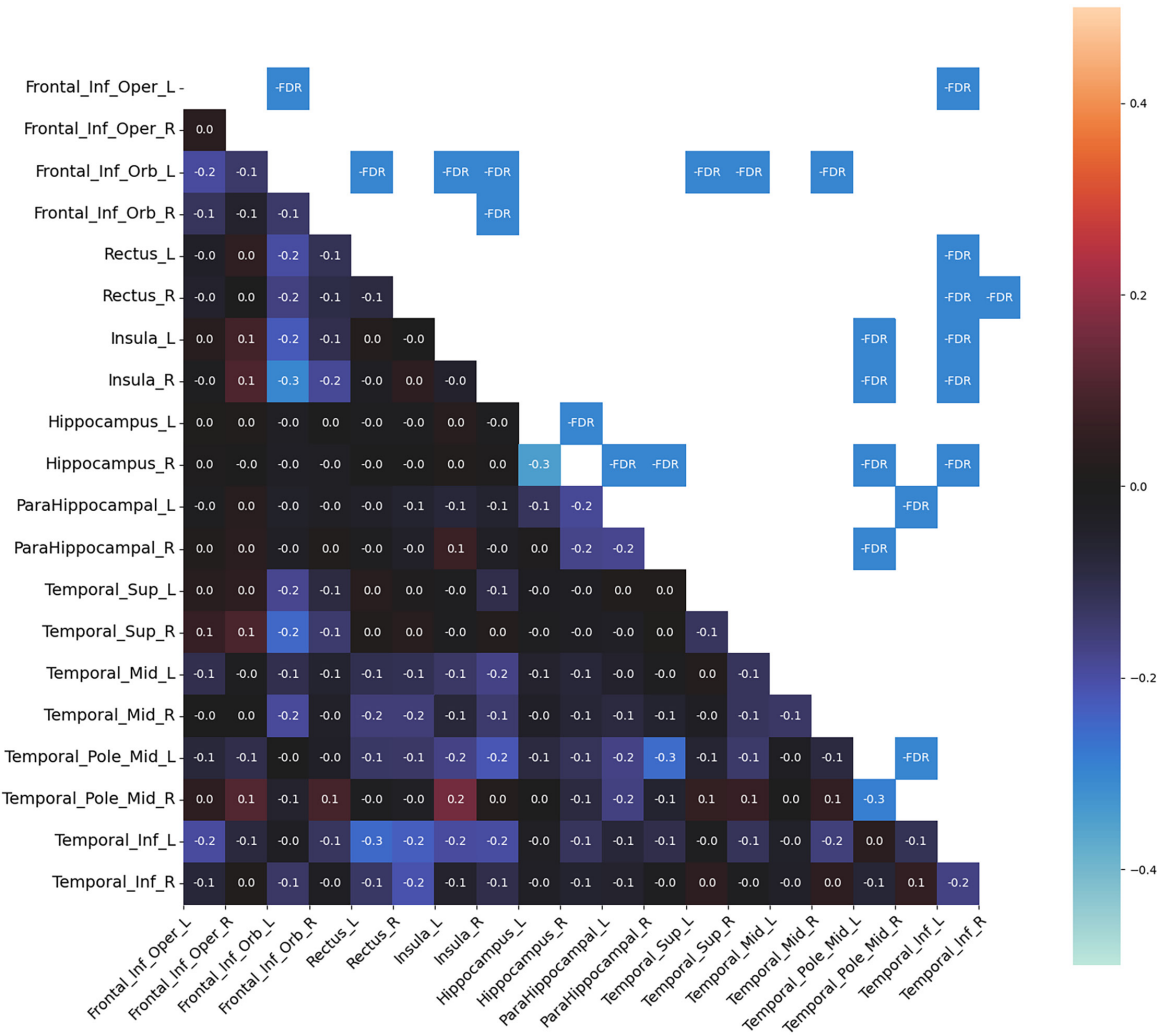
Metabolic connectivity (MC) changes in left hippocampal sclerosis. The differences in MC between healthy controls and left hippocampal sclerosis patients. The MC matrix in the lower left triangular graph represents the difference in strength connection between the corresponding regions of interest based on the AAL atlas. The warm/cold colors depict regions of interest pairs with significantly higher (warm colors) or lower (cold colors) MC in patients than in healthy controls. The upper right triangular graph shows statistically significant results. The significance level was set to p < 0.05, corrected for multiple testing (false discovery rate). FDR, false discovery rate; L, left; R, right.

**FIGURE 2:**
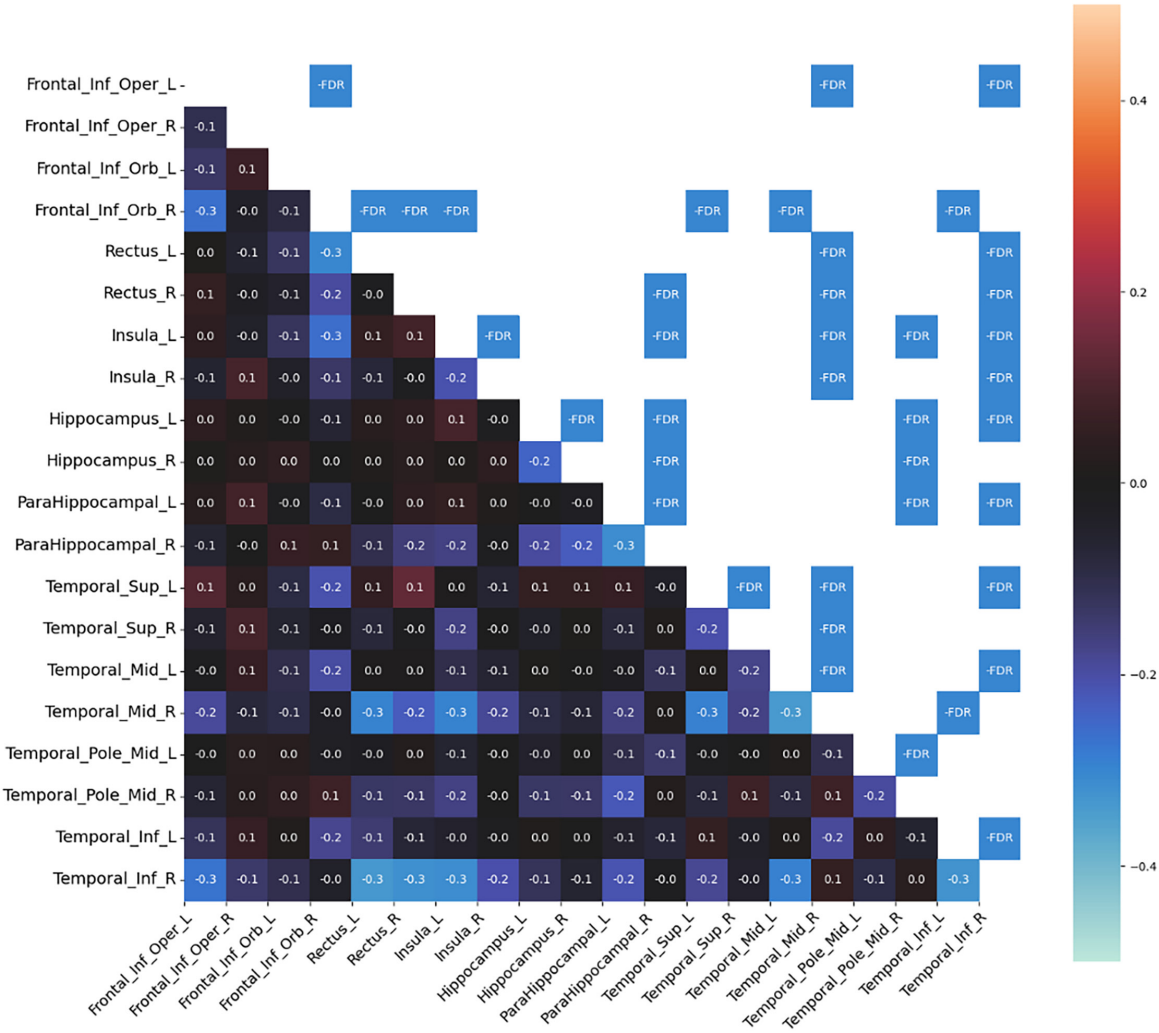
MC changes in right hippocampal sclerosis. The differences in MC between healthy controls and right hippocampal sclerosis patients. The MC matrix in the lower left triangular graph represents the difference in strength connection between the corresponding regions of interest based on the AAL atlas. The warm/cold colors depict regions of interest pairs with significantly higher (warm colors) or lower (cold colors) MC in patients than in healthy controls. The upper right triangular graph shows statistically significant results. The significance level was set to *p* < 0.05, corrected for multiple testing (false discovery rate). L, left; R, right; FDR, false discovery rate.

In general, more extensive MC changes were found in the right HS. The main difference was found in the ipsilesional temporal lateral cortex. Decreased MC between the ipsilesional lateral (and inferolateral) temporal cortex and other areas (both insulas, orbitofrontal cortices, and contralateral temporal cortex) was more pronounced in right HS than in left HS. None of the MC changes correlated with clinical features. Significant MC changes in left HS and right HS as compared with HC are shown in Figures 1 and 2.

### 3.3 Changes in MC in HS dependent on surgical outcome

#### 3.3.1 Decreased MC in non‐seizure‐free patients

Correlation of changes in MC with surgical outcome showed that non‐seizure‐free patients (ILAE 2–5) had significantly decreased MC between the lesional hippocampus, the ipsilesional frontal operculum and contralesional insula compared with seizure‐free patients (ILAE 1). There was also decreased MC between the contralateral hippocampus and the contralateral insula in non‐seizure‐free patients.

#### 3.3.2 Decreased MC in seizure‐free patients

**FIGURE 3:**
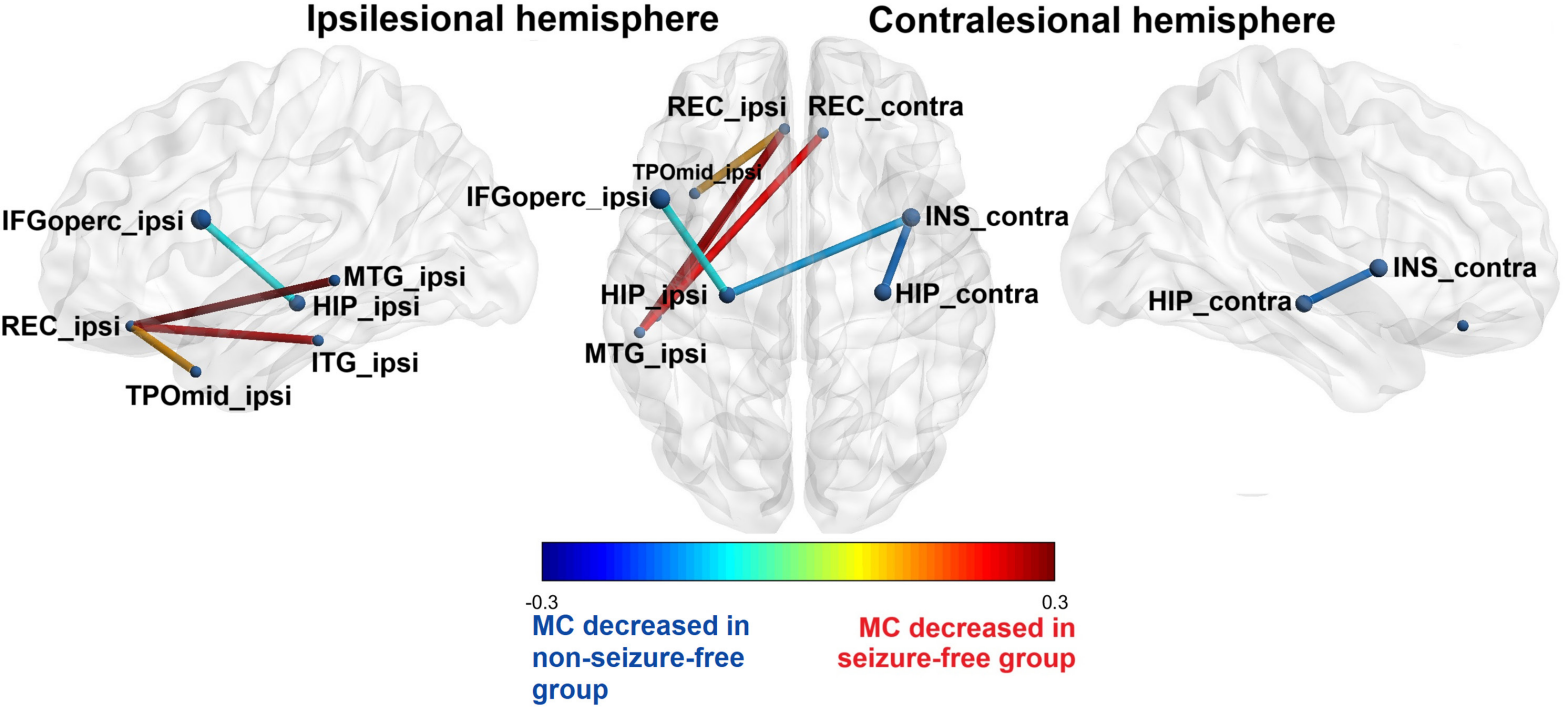
MC changes in hippocampal sclerosis correlated with surgical outcome. The significant differences in MC between seizure‐free (ILAE 1) and non‐seizure‐free (ILAE 2–5) patients with hippocampal sclerosis after surgery. Results after the unilateralization, depicted according to the side of the lesional hippocampus to the ipsilesional and contralesional side. Only statistically significant changes are shown. The warm/cold colors of lines between regions of interest that show significant changes of MC. Cold color depicts significantly decreased MC in non‐seizure‐free compared to seizure‐free patients. Warm color depicts significantly decreased MC in seizure‐free compared to non‐seizure‐free patients. The significance level was set to p < 0.05, corrected for multiple testing (false discovery rate). For illustration, the BrainNetViewer was used (http://www.nitrc.org/projects/bnv/).^37^ Contra, contralesional hemisphere; HIP, hippocampus; IFG, inferior frontal gyrus pars opercularis; INS, insula; Ipsi, ipsilesional hemisphere; ITG, inferior temporal gyrus; MTG, middle temporal gyrus; REC, gyrus rectus; TPOmid, temporal pole.

From another perspective, the seizure‐free patients showed significantly decreased MC (compared to the non‐seizure‐free patients) in both orbitofrontal cortices and the ipsilesional temporal lobe (temporal pole, inferior temporal cortex, and lateral cortex). Specifically, MC was decreased between the ipsilesional gyrus rectus and the ipsilesional temporal pole, the inferior and middle temporal gyrus, and between the contralesional gyrus rectus and the ipsilesional middle temporal gyrus. Significant changes in MC according to surgical outcome are summarized in Table 3 and Figure 3.

**TABLE 3:** Summary of results of intergroup metabolic connectivity correlated with surgical outcome.

Decreased MC	
I Hippocampus	I Frontal operculum (inferior frontal gyrus – pars opercularis)
	C Insula
C Hippocampus	C Insula
Increased MC	
I Lateral temporal cortex (middle temporal gyrus)	I Orbitofrontal cortex (gyrus rectus)
	C Orbitofrontal cortex (gyrus rectus)
I Inferolateral temporal cortex (middle temporal gyrus)	I Orbitofrontal cortex (gyrus rectus)
	C Orbitofrontal cortex (gyrus rectus)
Temporal pole (middle temporal gyrus – pole)	I Orbitofrontal cortex (gyrus rectus)

*Note*: The significant differences in metabolic connectivity between seizure‐free (ILAE 1) and non‐seizure‐free (ILAE non‐1) patients with hippocampal sclerosis after surgery. Results after the unilateralization, depicted according to the side of the lesional hippocampus to the ipsilesional and contralesional side. Metabolic connectivity between pairs of regions of interest is shown as significantly decreased or increased in the non‐seizure‐free group as compared to the seizure‐free group of patients.

Abbreviations: C, contralesional hemisphere; I, ipsilesional hemisphere; MC, metabolic connectivity.

#### 3.3.3 Correlation between MC and clinical variables

Decreased MC between the contralesional insula and hippocampus correlated with longer duration of epilepsy. Increased MC between the ipsilesional orbitofrontal cortex, temporal pole, inferior and middle temporal gyrus correlated with earlier epilepsy of onset. None of the MC changes correlated with electroclinical and structural characteristics. Details are shown in Table 4.

**TABLE 4:** Correlation of significant metabolic connectivity changes from surgical outcome point of view with clinical, electroclinical and structural characteristics.

MC	Pairs of ROIs		Epilepsy onset	Epilepsy duration	Seizure frequency	Electroclinical patterns	HS degree	Temporal atrophy	Polar signal abnormality
Decreased	I Hippocampus – I Frontal operculum (inferior frontal gyrus – pars opercularis)	*r*	0.19	−0.10	0.19	−0.03	−0.04	0.03	−0.01
		*p*	0.20	0.51	0.20	0.86	0.80	0.83	0.95
	I Hippocampus – C Insula	*r*	0.03	0.06	0.02	0.08	−0.08	0.00	−0.03
		*p*	0.84	0.69	0.90	0.60	0.58	0.98	0.83
	C Hippocampus – C Insula	*r*	−0.13	**0.29**	0.05	0.06	−0.04	−0.08	0.11
		*p*	0.38	**0.05**	0.74	0.70	0.79	0.59	0.46
Increased	I Lateral temporal cortex (middle temporal gyrus) – I Orbitofrontal cortex (gyrus rectus)	*r*	**0.29**	−0.26	−0.09	0.07	0.23	0.02	−0.11
		*p*	**0.05**	0.08	0.56	0.66	0.12	0.90	0.46
	I Lateral temporal cortex (middle temporal gyrus) – C Orbitofrontal cortex (gyrus rectus)	*r*	0.28	−0.28	−0.18	0.02	0.17	−0.05	−0.09
		*p*	0.06	0.06	0.22	0.88	0.26	0.74	0.53
	I Inferolateral temporal cortex (inferior temporal gyrus) – I Orbitofrontal cortex (gyrus rectus)	*r*	**0.31**	−0.19	−0.20	0.07	0.16	−0.03	−0.05
		*p*	**0.04**	0.21	0.18	0.64	0.29	0.87	0.76
	I Inferolateral temporal cortex (inferior temporal gyrus) – C Orbitofrontal cortex (gyrus rectus)	*r*	0.28	−0.17	−0.23	0.03	0.19	−0.04	−0.05
		*p*	0.06	0.25	0.12	0.86	0.19	0.80	0.71
	Temporal pole (middle temporal gyrus – pole) – I Orbitofrontal cortex (gyrus rectus)	*r*	**0.35**	−0.07	−0.20	0.00	−0.06	−0.09	−0.07
		*p*	**0.02**	0.63	0.17	1.00	0.70	0.54	0.65

*Note*: Correlation of the significant differences in metabolic connectivity between seizure‐free (ILAE 1) and non‐seizure‐free (ILAE non‐1) patients with hippocampal sclerosis after surgery with clinical, structural a electroclinical characteristics of the patients. Metabolic connectivity between pairs of regions of interest is shown as significantly decreased or increased in the non‐seizure‐free group as compared to the seizure‐free group of patients. Significant correlations are depicted in bold.

Abbreviations: C, contralesional hemisphere; I, ipsilesional hemisphere; MC, metabolic connectivity; *r*, Spearman's correlation coefficient; ROIs, regions of interest; *t*, *T*‐value.

## 4 DISCUSSION

This study examined the interictal metabolic network in patients with drug‐resistant MTLE with HS. The structure of the metabolic network was based on FDG–PET data. The study demonstrated the differences in MC between left HS and right HS and introduced MC as a predictive factor for surgical outcome. The evidence of the study is increased by the fact that only a homogeneous group of patients with histopathologically proven HS was included and the study had a relatively long follow‐up period. From a practical point of view, the study focused directly on the cortical areas adjacent to the mesiotemporal structures and the areas of MTLE seizure spread as potential targets for more precise presurgical evaluation in selected patients.

Hypometabolism is typical of the epileptogenic zone. This may result from neuronal loss or from inhibitory processes; the exact mechanism is unknown.^16^, ^17^, ^25^ Changes in FDG metabolism have been observed in the epileptogenic hippocampus in the surrounding area and also extratemporally. According to PET studies, metabolic changes in HS may correlate with clinical features, such as the age at seizure onset, duration of epilepsy which was also found in our study.^17^, ^26^ We did not find the correlation between MC and seizure frequency. Metabolic changes also correlate with surgical outcome and lateralization of the epileptogenic hippocampus, which was also confirmed in our study in terms of connectivity.^17^

### 4.1 MC in HS

Both patients with left HS and right HS showed decreased MC between bilateral mesiotemporal structures (hippocampus or parahippocampal gyrus), probably reflecting the long‐term effects of the epileptogenic hippocampus on the non‐affected hippocampus, as other studies^24^, ^27^ have shown that bitemporal PET hypometabolism correlates with epilepsy duration. Decreased MC was also found in other areas, typically in the lesional hemisphere: temporal pole, lateral temporal cortex, orbitofrontal cortex, and especially in the inferior temporal gyrus. These are all areas of decreased metabolism in HS.^17^, ^20^, ^24^ In the contralateral hemisphere, most changes were found in the insula, orbitofrontal cortex, and temporal lateral cortex. The finding of an altered metabolic network reaching these contralesional areas is novel. However, it is not surprising as these are typical areas for the propagation of ictal activity in MTLE seizures and supports the findings of the FC studies showing widespread bilateral changes in patients with HS.^28^

### 4.2 Different MC patterns in left and right HS

Our results showed that the changes in MC were more pronounced in right HS than in left HS. This asymmetry between left and right HS cannot be explained by asymmetric clinical features, as there were no significant differences in clinical features between these groups. Metabolic asymmetry in HS is a well‐known phenomenon. The largest PET study investigating left HS and right HS separately showed that the metabolic changes (hypometabolism or hypermetabolism) are more widespread in right HS, which is in agreement with our study.^17^ However, another study showed the opposite results, with greater metabolic changes in left HS.^29^ Functional network changes are typically greater in the left HS.^10^, ^11^ Regarding changes in MC, no study has shown such an asymmetry because previous studies have not compared left and right HS separately.^8^, ^9^ Compared to left HS, the most significant decrease in MC in right HS was found in the ipsilesional temporal lateral (especially inferolateral) cortex; other findings were bilateral insulas, orbitofrontal cortices, and contralateral temporal cortex. This is consistent with PET and MRI studies showing more pronounced changes in some of these regions in the right HS, but the clinical significance of these findings is unclear.^12^, ^17^ We did not find a worse surgical outcome in RTLE than in LTLE. Therefore, the clinical impact of more pronounced changes in the right HS is uncertain. In the unilateralized PET data, we found that surgical outcome correlated with decreased FC within a network of the hippocampi and operculoinsular regions. We could assume that the MC of these areas was not altered in RTLE (compared with LTLE) enough to affect the outcome.

### 4.3 Correlation of metabolic network with surgical outcome

Comparing non‐seizure‐free patients (ILAE 2–5) with seizure‐free patients (ILAE 1) after surgery, unfavorable surgical outcomes correlated with significantly decreased MC of the lesional hippocampus and both the ipsilateral frontal operculum and the contralateral insula. It also correlated with decreased MC between the contralateral hippocampus and the contralateral insula. The insula is a typical area for the propagation of temporal seizures and PET hypometabolism in HS.^17^, ^24^ PET studies have shown that both ipsilesional and contralateral insula hypometabolism may be markers of unfavorable outcomes.^7^, ^20^ However, an SEEG study showed that insula involvement in temporal seizures was not a negative prognostic factor for surgical outcomes.^30^ The change in MC could be a consequence of the preferential spread of epileptic activity or it could reflect the existence of a temporo‐operculo‐insular epileptogenic network in HS. The involvement of the contralateral hippocampus in the pathological metabolic network is predictable, as contralateral temporal hypometabolism has already been identified as a negative prognostic factor.^20^ Such hypometabolism could also reflect the spread of epileptic activity contralaterally or the possible presence of bitemporal epilepsies among our patients.^24^ Analogously, all of these changes in the metabolic network in non‐seizure‐free patients, extending beyond the pathological hippocampus to the orbitofrontal cortex and operculo‐insular region, could also indicate the presence of temporal‐plus or temporal neocortical epilepsy in our patients with MTLE.^31^ Our study shows that both the hippocampi and the operculo‐insular regions could be part of a pathological metabolic network, probably reflecting a long‐term preferential spread of epileptic activity. We hypothesize that standard AMTR did not sufficiently disrupt this network and thus caused the failure of the surgery. This hypothesis of long‐term consequences of preferential spreading is supported by the finding that MC changes in the contralesional insula and hippocampus correlate with a longer duration of epilepsy. It also highlights the importance of early surgery in patients with DRE with HS to prevent possible pathological network changes.

A favorable surgical outcome correlated with a relatively decreased MC in non‐seizure‐free patients (compared to seizure‐free patients) between both orbitofrontal cortices and the ipsilesional temporal lobe (temporal pole, temporal inferior, and lateral cortex). The correlation between relative hypometabolism and positive surgical outcomes in focal epilepsy is problematic because hypometabolism is the usual marker of an epileptogenic focus.^5^, ^7^, ^32^, ^33^ A better outcome has been described with relative hypometabolism in the ipsilesional orbitofrontal cortex and temporal pole. Dupont hypothesized that the hypometabolism of these structures reflects a preferential path for seizure propagation to these structures. After AMTR, this pathway is disrupted and therefore these patients are seizure‐free.^5^ We confirm this hypothesis but add the temporal lateral and inferolateral temporal cortex involvement to this network. We also found that these changes are probably a long‐term effect of such a preferential spread, as it positively correlates with the earlier epilepsy onset.

MC changes relevant to surgical outcome did not correlate with electroclinical patterns. According to PET studies, hypometabolism may differ between different electroclinical patterns.^7^, ^24^ The non‐significant correlations in our study may be due to the small sample size. An alternative explanation could be that MC reflects more network changes than hypometabolism alone, making comparison with FC studies more appropriate. The correlation between semiology and network (FC changes) is problematic and still a matter of research.^34^ We also found no correlation of MC changes relevant to surgical outcome with any of the structural characteristics. This result supports the findings of FC studies that network changes may be independent of structural abnormalities.^35^, ^36^

## 5 CONCLUSION

Metabolic network changes extend beyond areas traditionally described as having altered metabolism in HS, in particular the contralateral orbitofrontal cortex. Metabolic connectome changes in left HS are more extensive than in right HS, especially in the ipsilesional temporal lateral cortex. MC alterations may serve as prognostic factors in MTLE with HS. Unfavorable outcomes are found in patients with decreased MC within the network including both hippocampi and operculoinsular regions. The results indicate the presence of a pathological temporo‐operculo‐insular metabolic network that could generate seizures even after standard AMTR. Furthermore, the favorable outcomes correlate with decreased MC within the network, which includes both the orbitofrontal cortices and the ipsilateral temporal lobe. Some of the MC changes relevant to surgical outcomes correlate with earlier onset or longer epilepsy duration. With regard to the study results, we emphasize the importance of advanced processing of PET data in patients with HS during preoperative assessment. MC can identify patients with potentially unfavorable outcomes and lead them to more detailed investigations such as invasive EEG. Possible targets for such investigations should include the orbitofrontal, temporal lateral, and operculo‐insular areas in addition to both hippocampi. However, the relationship between metabolic and epileptogenic networks should be the subject of further research.

## AUTHOR CONTRIBUTIONS

Ondřej Strýček: Analysis of literature, design of the study, and writing and approval of the manuscript. Pavel Říha: Data analysis, writing, and approval of the manuscript. Zdeněk Řehák: Data acquisition, approval of the manuscript. Marin Kojan: Design of the study, data analysis, writing, and approval of the manuscript. Milan Brázdil: Review and critique.

## FUNDING INFORMATION

Supported by project nr. LX22NPO5107 (MEYS): Financed by European Union – Next Generation EU. Supported by MH CZ – DRO (MMCI, 00209805).

## CONFLICT OF INTEREST STATEMENT

None of the authors has any conflict of interest to disclose. We confirm that we have read the Journal's position on issues involved in ethical publication and affirm that this report is consistent with those guidelines.

## Data Availability

The datasets used and analyzed during the current study are available from corresponding author on request.
